# Congenital Lung Malformations: A Pictorial Review of Imaging Findings and a Practical Guide for Diagnosis

**DOI:** 10.3390/children11060638

**Published:** 2024-05-25

**Authors:** Giovanna Cancemi, Giulio Distefano, Gioele Vitaliti, Dario Milazzo, Giuseppe Terzo, Giuseppe Belfiore, Vincenzo Di Benedetto, Maria Grazia Scuderi, Maria Coronella, Andrea Giovanni Musumeci, Daniele Grippaldi, Letizia Antonella Mauro, Pietro Valerio Foti, Antonio Basile, Stefano Palmucci

**Affiliations:** 1 U.O.C. Radiodiagnostica Lentini, ASP Siracusa, 96016 Siracusa, Italy; cancemi.giovanna@gmail.com; 2 Institute of Nephrology and Dialysis—Nephrological, Vascular and Internal Medicine Diagnostic Ultrasound Service, Maggiore Hospital of Modica, ASP Ragusa, 97015 Modica, Italy; giulio.distefano@asp.rg.it; 3 Department of Medical Surgical Sciences and Advanced Technologies “GF Ingrassia”, University Hospital Policlinico G. Rodolico-San Marco, University of Catania, 95123 Catania, Italy; vitalitig@gmail.com (G.V.); dario.milazzo2015@libero.it (D.M.); drgiuseppeterzo@hotmail.com (G.T.); giuseppebelfiore18560@gmail.com (G.B.); mcoronella@sirm.org (M.C.); pietrovalerio.foti@unict.it (P.V.F.); basile.antonello73@gmail.com (A.B.); 4 Division of Pediatric Surgery, University Hospital Policlinico G. Rodolico-San Marco, University of Catania, 95123 Catania, Italy; vdbchirurgiapediatrica@gmail.com (V.D.B.); mgscuderi@gmail.com (M.G.S.); 5 Casa di Cura Regina Pacis, 93017 San Cataldo, Italy; andreagiovannimusumeci@gmail.com; 6 UOSD I.P.T.R.A.-Department of Medical Surgical Sciences and Advanced Technologies “GF Ingrassia”, University Hospital Policlinico G. Rodolico-San Marco, University of Catania, 95123 Catania, Italy; grippaldidaniele@gmail.com (D.G.); mauroletizia@tiscali.it (L.A.M.)

**Keywords:** congenital lung malformations, congenital thoracic malformations, congenital lung anomalies, imaging evaluation, imaging guidelines, computed tomography, radiography, MR imaging, ultrasound

## Abstract

The term congenital lung malformation (CLM) is used to describe a wide range of pathological conditions with different imaging and clinical manifestations. These anomalies stem from abnormal embryological lung development, potentially occurring across various stages of prenatal life. Their natural history can be variable, presenting in a wide range of severity levels and encompassing asymptomatic individuals who remain so until adulthood, as well as those who experience respiratory distress in the neonatal period. Through the PubMed database, we performed an extensive review of the literature in the fields of congenital lung abnormalities, including their diagnostic approach and findings. From our RIS-PACS database, we have selected cases with a final diagnosis of congenital lung malformation. Different diagnostic approaches have been selected, including clinical cases studied using plain radiograph, CT scan, prenatal ultrasound, and MR images. The most encountered anomalies can be classified into three categories: bronchopulmonary anomalies (congenital pulmonary airway malformations (CPAMs), congenital lobar hyperinflation, bronchial atresia, and bronchogenic cysts), vascular anomalies (arteriovenous malformation), and combined lung and vascular anomalies (scimitar syndrome and bronchopulmonary sequestration). CLM causes significant morbidity and mortality; therefore, the recognition of these abnormalities is necessary for optimal prenatal counseling and early peri- and postnatal management. This pictorial review aims to report relevant imaging findings in order to offer some clues for differential diagnosis both for radiologists and pediatric consultants.

## 1. Introduction

Congenital lung malformation (CLM) is a term encompassing a wide variety of disorders of the large airways, lung tissue, and pulmonary blood vessels, including what was once termed congenital pulmonary airways malformation (CPAM), as well as intra- and extra-lobar pulmonary sequestration (PS), congenital large hyperlucent lobe (CLHL, also known as congenital alveolar overdistension or congenital lobar emphysema), bronchogenic cysts, and bronchial atresia [1].

According to published studies, CLMs account for 5–18% of all congenital abnormalities, with a cumulative incidence of 30–42 cases per 100,000 individuals. Consequently, they are considered rare disorders [1]. However, the overall incidence of CLMs has increased compared to the past two decades, likely due to the routine use of prenatal ultrasound, and these anomalies are often detected “in utero” [2].

**Figure 1:**
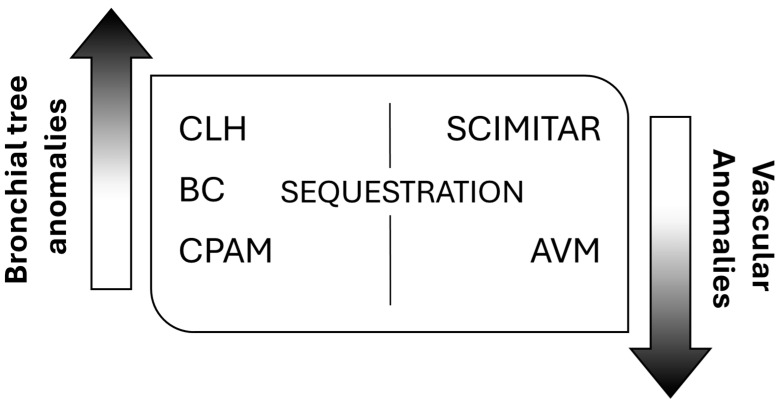
General classification of congenital lung malformations (CLMs). CLH: congenital lobar hyperinflation; BC: bronchogenic cyst; CPAM: congenital pulmonary airway malformation; AVM: arteriovenous malformation.

Despite multiple publications on the topic, no structured system of nomenclature exists to describe all lung abnormalities. They can manifest as a pure lung parenchymal abnormality with normal vasculature (e.g., congenital pulmonary airway malformation, CPAM), as a pure vascular abnormality with normal lung (e.g., pulmonary arteriovenous malformation, AVM), or as a combination of both parenchymal and vascular abnormalities (e.g., bronchopulmonary sequestration and hypogenetic lung or scimitar syndrome, which is part of congenital pulmonary venolobar syndrome) [3]. According to this, the most frequently encountered anomalies fall into three main categories, bronchopulmonary anomalies, vascular anomalies, and combined lung and vascular anomalies [4], spanning a continuum of maldevelopment [5] (Figure 1).

Although the pathological classification of CLMs can be established only through histological examination, it is mandatory to describe each phenotypic feature of these entities, because more than one of these anomalies may be present in the same case [1]. However, recognizing these anomalies antenatally can provide early management and help in providing parental counseling, including pregnancy decisions and surgical planning.

The objective of this pictorial review is to establish a comprehensive classification of the most frequently observed congenital lung anomalies, emphasizing significant imaging features that are helpful for differential diagnosis.

## 2. Materials and Methods

Through the PubMed database, an extensive review of the existing literature was performed in the fields of congenital lung malformations, including their diagnostic approach and imaging findings. We used the following keywords: “congenital lung malformations”, “congenital thoracic malformations”, and “congenital lung anomalies”. We combined these keywords with the following: “imaging evaluation”, “imaging guidelines”, “computed tomography”, “radiography”, “MR imaging”, and “ultrasound”. 

Our keyword research did not specify any interval in the search period. We exclusively considered articles in English, ensuring access to their entire content. Articles in other languages and recurrent articles from the same authors were excluded from our analysis. Information deemed relevant was extracted from original articles, reference guidelines, and previous reviews. The title, abstract, and bibliography of the articles were then analyzed to further evaluate the suitability of the publications for this review. Publications deemed to be pertinent to the review’s objective were then selected.

Regarding images’ collection, we selected radiological cases from our RIS-PACS system with a final diagnosis of congenital lung malformation, concerning a period ranging from November 2019 to March 2024. Postnatal cases were routinely evaluated on neonatal plain radiograph and chest CT. The images were archived in accordance with the Laws of the State and the internal regulations of our Institute. 

A preliminary review of the axial and multiplanar reconstructed CT images, prior to lung resection or treatment, has been performed by board-certified and fellowship-trained radiologists, using a picture archiving and communication system (PACS).

For the purposes of this review, it should first be noted that all data pertaining to prenatally diagnosed lesions have been excluded, on account of the data having been collected by obstetric colleagues during the initial ultrasound.

## 3. Results and Discussion

### 3.1. Etiopathogenesis of CLMs

Several hypotheses regarding the etiology of CLMs have been proposed, but comprehending the pathogenesis remains challenging [1]. 

The prevailing theory proposes that congenital lung anomalies arise from the flawed budding of the tracheobronchial tree from the primitive foregut at various stages of intrauterine life, generally occurring between days 24 and 36 of gestation [1,3]. Both the type and histopathology of these anomalies are believed to be correlated with the timing of the embryologic insult [6]. While this theory could elucidate congenital foregut duplication cysts, it does not as readily account for other forms of CLMs. Another theory proposed that the cause of these abnormalities may be the inhibition of the complex interaction between the developing lung bud and its surrounding mesenchyme, caused by infection, vascular interruption, or airway obstruction [7]. The underlying mechanism resulting in secondary pulmonary dysplastic changes is mainly based on two parameters: the severity and timing of airway obstruction development (whether complete or partial) and the origin of the foregut airway bud (normal or ectopic) [7]. According to this, a complete occlusion at an early stage is likely to result in a failure of lung or lobes development, whereas a more delayed occlusion may give rise to a distal dysplasia, observed in cases of CPAM, intra-lobar sequestration, and bronchial atresia. The partial obstruction to the developing airway is thought to lead to distal air trapping and dysplasia, conditions observed in congenital lobar emphysema [7]. Bronchogenic cysts and extra-lobar sequestration share a common pathological mechanism. They differ, however, in that the foregut airway buds arise in an ectopic location [7]. This theoretical framework can also elucidate the coexistence of distinct entities in a single congenital lung malformation. Examples include bronchial atresia concomitant with pulmonary sequestration and hybrid lesions, which exhibit characteristics of both CPAM and sequestrations [7].

Another theory proposed that vascular abnormalities may be implicated in the pathogenesis of congenital lung anomalies; this theory helps to explain combined lung and vascular anomalies, as seen in cases such as congenital lung hypoplasia associated with a pulmonary artery sling. However, this theory does not fully account for the presence of pure pulmonary parenchymal anomalies [8]. 

A more recent hypothesis regarding the pathogenesis of lung malformations suggests that an underlying genetic cause may be responsible. According to a previously published study, the excessive signaling of fibroblast growth factor 10 (FGF10) may cause the formation of abnormal cystic-like structures during early lung development [6]. In a subsequent study, it was demonstrated that individuals exhibiting abnormalities within their airways showed elevated expression levels of epithelial airway markers in addition to dysregulated gene expression patterns within the Ras and several kinases signaling pathways [9]. Furthermore, a study conducted on mice has revealed that mutations in the DICER1 gene can lead to the formation of cystic airways, the disruption of branching morphogenesis, and mesenchymal expansion, which are similar to the characteristics observed in pleuropulmonary blastomas (PPBs) [10].

### 3.2. Clinical Manifestations and Complications Associated with CLMs

Most CLMs are detected “in utero” [11]. The solid or cystic lesion may undergo enlargement, resulting in a mediastinal shift, or may regress completely or partially prior to birth [1,12,13]. The most serious complication of pregnancy is fetal hydrops. This occurs in 3–5% of cases and often necessitates prompt prenatal intervention and premature delivery [1,14]. Other fetal complications include pleural effusion, or polyhydramnios, which results from esophageal compression and the subsequent failure of normal fetal swallowing [1]. The natural history of CLMs can vary considerably at birth. During the early neonatal period, some lesions may determine the compression of the surrounding parenchyma, leading to respiratory distress; conversely, some individuals may remain asymptomatic, and CLMs can only be diagnosed on a conventional chest X-ray performed for reasons unrelated to the disease [15].

In the neonatal period, most newborns (>75%) are asymptomatic, with only a small percentage of them necessitating any respiratory support [1]. The clinical presentation in neonates consists of recurrent wheeze or chronic cough and infections around the age of 7 months on average [1,14]. In some cases, the coexistence of extrapulmonary anomalies, such as congenital diaphragmatic hernia, often associated with PS, as well as cardiovascular anomalies, may suggest the presence of CLMs [1,16,17]. Postnatal complications of misdiagnosed or untreated CLMs include recurrent pneumonia (both bacterial and fungal), hemoptysis and/or hemothorax, pneumothorax, air embolism, potential tumor development, and even heart failure as a result of systemic collateral shunting [7,16,18]. 

At the adult age, some lesions may be asymptomatic and observed incidentally on chest imaging. Others are associated with a range of symptoms, including cough, hemoptysis, recurrent pneumonia, dysphagia, and chest pain [19,20]. 

The association between congenital pulmonary airway malformations (CPAMs) and malignancies remains a subject of controversy, indicating the unresolved nature of this issue—which requires further investigation. Evidence suggests that CPAMs may elevate the risk of developing certain malignant neoplasms, including pleuropulmonary blastoma (PPB), bronchioloalveolar carcinoma, and lung adenocarcinoma [21,22,23]. PPB, in particular, is a rare tumor, with an incidence of 1 in 250,000 live births, which rises to approximately 4% in children with CPAM. However, the relationship between CPAM and PPB remains contentious [10]. It remains unclear whether type 4 CPAM represents a regressed type 1 PPB or if type 1 PPB arises as a consequence of type 4 CPAM. One hypothesis posits that the cystic component may result from tumor necrosis, while an alternative theory suggests that the tumor may develop within cysts. Finally, PPB has been identified as a distinct entity, exhibiting imaging features similar to those of CPAM; in particular, clinical and radiological features that are predictive of PPB include the presence of symptoms (particularly pneumothorax), bilateral or multisegmented involvement, the presence of a complex cyst, and a germline mutation in the DICER1 gene, found in two thirds of PPB cases [10,23,24,25]. Furthermore, the possible malignant transformation of CLMs has been investigated. 

A recent study on gene expression in CPAM lesions demonstrated that KRAS mutations and MUC5AC, CK20, and HER2 expression was seen in all CLMs with mucinogenic proliferations, supporting the possible neoplastic nature of type 1 CPAM. These findings emphasize the importance of the complete surgical resection of these CLMs [26]. Type 1 CPAM presents with intra-cystic mucinous cell clusters, which may form extra-cystic mucinous proliferation resembling mucinous bronchioloalveolar carcinomas, of which it may be the precursor [27]. A more recent study reported an association with lung rhabdomyosarcoma in addition to PPB and that bronchioloalveolar carcinomas or adenocarcinoma occurred more often in the adult population in association with BC [28]. This study suggests that the connection between congenital lung malformations (CLMs) and lung tumors is likely incidental. Furthermore, it indicates that various types of CLMs, including congenital pulmonary airway malformation type 1 (CPAM1) and bronchogenic cysts (BC), may be linked with malignant lung lesions, with CPAM1 being the most frequently associated. These tumors can arise at any age. Consequently, a surgical approach may be considered an acceptable option in light of the potential risk of the malignant transformation of CLMs [28].

### 3.3. Diagnostic Approach

Actually, there is no uniform diagnostic approach, so clinicians must rely on prenatal ultrasound, the initial postnatal lung imaging findings (principally chest X-ray findings), and any associated clinical features. Various diagnostic techniques, including prenatal ultrasound (US), chest radiography, computed tomography (CT), and magnetic resonance (MR) have been used to suggest a simplified algorithm for differential diagnosis.

In the antenatal period, serial fetal ultrasound is the investigation that is typically performed to evaluate parenchymal lung anomalies, such as the location, size, and appearance of the lesion (solid, macro/micro-cystic, or mixed lesion), as well as blood supply, mediastinal shift, pleural effusion, or fetal hydrops [16]. While the regression of the lesion is a common occurrence during the third trimester, it is advisable to consider intervention if the lesion shows progressive enlargement until the last 10 weeks of pregnancy or if hydrops is present [16]. Fetal MRI, in addition to US, may serve as a valuable adjunct diagnostic tool in the identification of a systemic arterial blood supply and the detection of associated complications [29]. The CLM volume ratio (CVR) is a prognostic tool that can be used to predict the risk of fetal hydrops or respiratory distress at birth in cases of cystic lesions. This ratio is calculated as the CPAM lesion length, width, and height (in cm²) multiplied by 0.52 and divided by the head circumference in cm². A CVR value of ≥0.84 is a reliable predictor of respiratory distress at birth [30]. Moreover, an increase in the cardio-mediastinal shift angle has been demonstrated to be significantly associated with an unfavorable perinatal outcome in the case of CLMs [31]. 

Following birth, although the majority of congenital lung lesions are identified during prenatal US, chest radiography is typically the initial imaging modality employed to characterize these lesions. The imaging characteristics of congenital lung anomalies vary according to their specific type and size. However, plain radiograph may offer some specific clues for diagnosis such as thoracic asymmetry, focal mass/consolidation, focal hyperlucency, airway abnormalities, vascular abnormalities, and other lesions, including vertebral anomalies, gastrointestinal anomalies, and cardiac anomalies [8,32]. 

Given the low sensitivity of chest radiography [33], it is advisable to perform a CT scan during the first 6 months of life to confirm the presence of suspected CLMs. When conducting the CT scan, it is advised to adjust the milliamperage and kilovoltage settings based on the patient’s age or weight to minimize radiation exposure [32]. Additionally, CT 3D reformations of the lung, airway, and vascular structures are indispensable in the evaluation of both pathological parenchyma and vessels, as well as for the preoperative assessment [15,32]. Many authors have proposed the utilization of contrast prior to surgery due to its potential in evaluating the vascular supply, especially if PS is suspected [15,32,34]. 

While postnatal magnetic resonance imaging (MRI) may not be ideal for evaluating subtle lung parenchymal abnormalities, it offers a compelling alternative to computed tomography (CT) by eliminating ionizing radiation exposure. Furthermore, MRI demonstrates superior sensitivity compared to CT when assessing the vascular anatomy, particularly for small vessels situated near the cardiac cavity [34,35]. 

In some patients, especially if asymptomatic, the diagnosis may remain unknown and only detected in adulthood chest x-ray performed for other reasons [1].

### 3.4. Bronchopulmonary Anomalies

#### 3.4.1. Pulmonary Underdevelopment

Pulmonary underdevelopment has been categorized into three distinct groups based on their radiological characteristics [3]. The term pulmonary “agenesis” indicates the total absence of lung tissue, including both bronchi and vasculature [35]. Radiological findings in pulmonary agenesis and “aplasia” are akin, save for the existence of a blind-ending rudimentary bronchus in pulmonary aplasia [3,35]; prenatal ultrasound struggles to ascertain this condition definitively, often raising suspicion based on mediastinal displacement [36]. Chest radiograph demonstrates an opacified hemithorax associated with a marked ipsilateral mediastinal shift, and CT confirms the absence of lung parenchyma and blood supply, often accompanied by ipsilateral osseous structures’ hypoplasia [3].

Furthermore, the term “hypoplasia” is related to the presence of a bronchus and rudimentary lung; also, the airways, alveoli, and pulmonary vessels are reduced in size and number [3,37]. This condition is typically secondary to a process (intra- or extra-thoracic), which is most commonly a congenital diaphragmatic hernia. This process leads to a reduction in the intrathoracic space, which in turn results in pulmonary maldevelopment [3]. In this case, liver herniation in prenatal US can be confused with a solid mass originating in the lung; color doppler may help to identify portal and hepatic veins, despite the fact that MR imaging is more sensitive in detecting liver herniation [3,38]. 

Other intrathoracic causes of pulmonary hypoplasia include CPAM, bronchopulmonary sequestration, a cardiac or mediastinal mass, lymphatic malformation, and the agenesis of the diaphragm [3,37]. The leading extrathoracic cause is often severe oligohydramnios, typically resulting from genitourinary anomalies—which include renal agenesis or severe renal dysfunction—or resulting from the prolonged preterm rupture of membranes [3,37]. Chest radiographs may reveal a small lung and retrosternal hyperclarity in cases of pulmonary hypoplasia, attributed to the herniation of the contralateral lung [39]. However, the imaging modalities of choice to discriminate between lung agenesis, aplasia, and hypoplasia are CT and MRI, allowing the differentiation of the absence or hypoplasia of the lung parenchyma, bronchial tree, and pulmonary vessels [37].

#### 3.4.2. Congenital Pulmonary Airway Malformations

Congenital pulmonary airway malformations (CPAMs) are a heterogeneous group of cystic and non-cystic lung lesions that result from the disorganized proliferation of different structures of the tracheo-bronchial tree. The term CPAM is preferable to the term congenital cystic adenomatoid malformation (CCAM), since the lesions are cystic in only three of the five types of these lesions and adenomatoid in only one type (type 3). CPAMs were first classified by Stocker according to cyst size and histology [40]. The newer classification into five types is an extension of the previous one (that included three types) [41] (Table 1).

**Figure 2:**
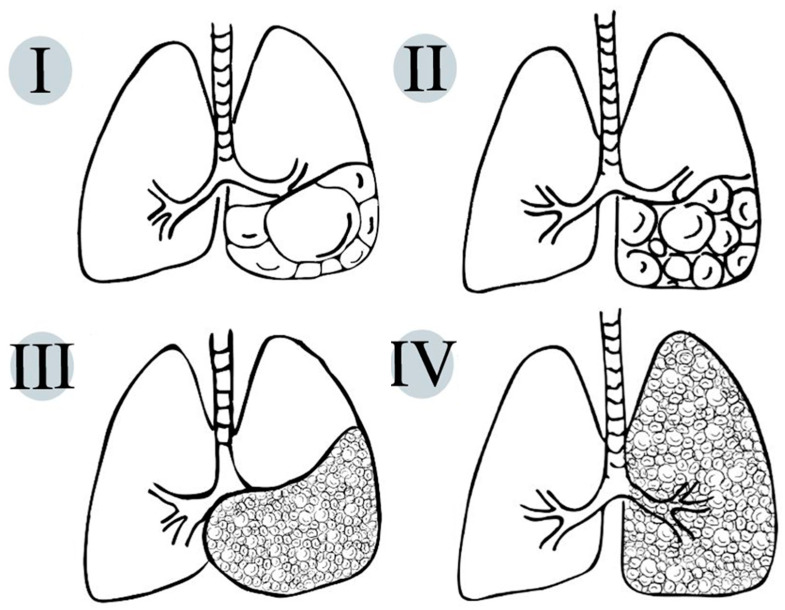
Schematic representation of life-compatible types of congenital pulmonary airway malformations, which characteristics are thoroughly described in Table 1.

On pathologic examination, CPAMs show the exuberant overgrowth of the primary bronchioles and can present communication with an abnormal bronchial tree (Figure 2).

In the majority of cases, the blood supply originates from the pulmonary artery, with venous drainage directed toward the pulmonary veins [3,8,32]. However, hybrid lesions, which are defined as a combination of congenital pulmonary airway malformation (CPAM) and pulmonary sequestration, may exhibit a systemic blood supply [37]. These histological findings imply the hypothesis that CPAMs could result from intrauterine airway obstruction [8].

Individuals with CPAM generally manifest respiratory distress early in infancy. In older children or adults, recurring infections—frequently depicted in the same lung region—can be observed on imaging. It should be noted that CPAMs may not be symptomatic during childhood but may later be discovered as incidental findings in adults [15]. Most CPAMs are detected during prenatal US in asymptomatic patients, appearing as numerous variable-sized anechoic spaces inter-mixed with echogenic soft tissue [3,32]. 

Postnatally, imaging may recognize only three types of CPAMs: large cysts CPAM (type 1), small cysts CPAM (type 2), and solid or microcystic type (type 3). Type 0 is incompatible with life (so it is not depicted on radiological images), while type 4 may be indistinguishable at imaging from a cystic pleuropulmonary blastoma [3,32].

**Figure 3:**
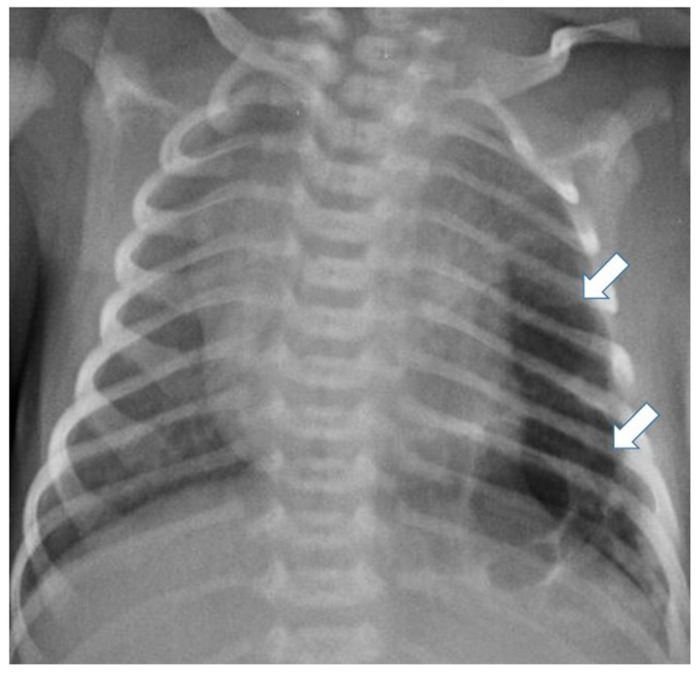
Chest radiograph of a newborn shows multiple large air-filled cysts in the left lower lobe (arrows), suggesting a type 1 CPAM.

**Figure 4:**
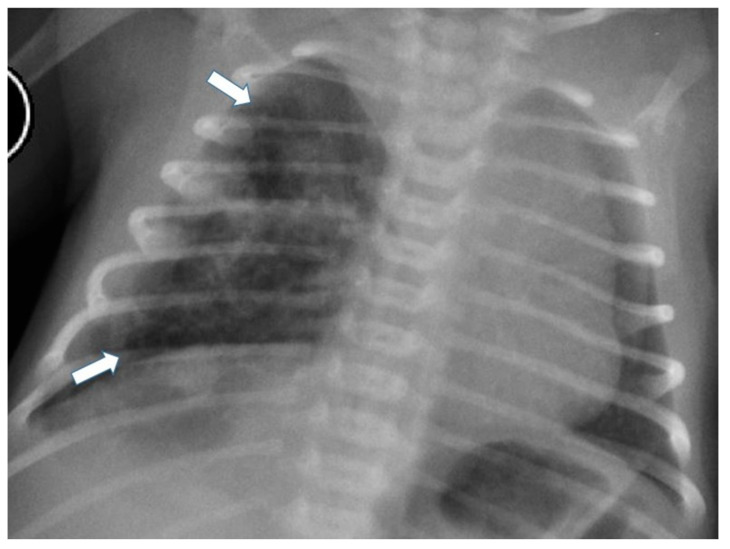
Chest radiograph of a newborn shows multiple small air-filled cysts in the right upper lobe and right lower lobe (arrows), suggesting a type 2 CPAM.

Postnatal chest radiography shows a region of variable opacity, dependent on the solid component of the lesion and the fluid contents of the cysts; a mediastinal shift may be associated [3,8] (Figure 3 and Figure 4).

**Figure 5:**
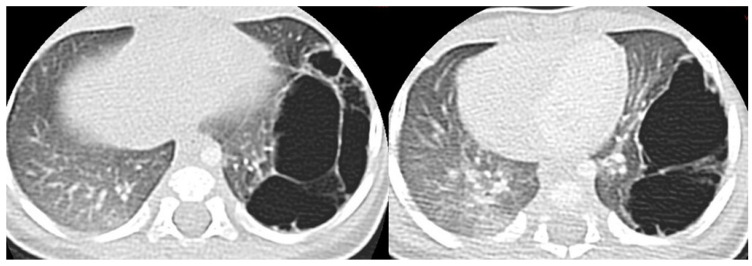
A CT scan performed one month later in the same patient as in Figure 3, showing multiple large air-filled cysts involving the left lower lobe, with a maximum diameter of 60 mm, confirming the diagnostic hypothesis of type 1 CPAM.

**Figure 6:**
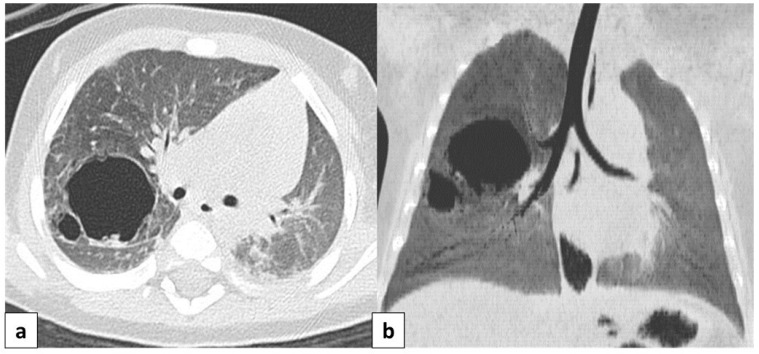
Axial CT scan performed on a 4 month old female patient, depicting a type 1 CPAM. In image (**a**), it is possible to appreciate a large (>2 cm) air-filled cyst, surrounded by other multiple smaller cysts. Additionally, in image (**b**), a coronal CT MinIP reconstruction of the same patient is shown, which excludes communication with the tracheo-bronchial tree.

**Figure 7:**
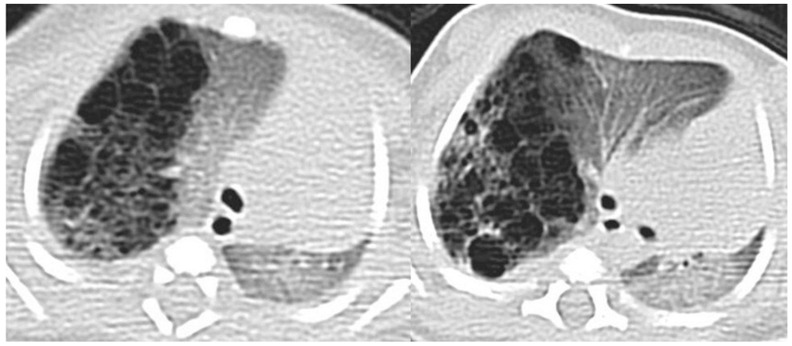
A CT scan later performed in the same patient as in Figure 4, showing multiple small air-filled cysts in the right upper lobe and in the right lower lobe, with a maximum diameter of 20 mm, confirming the diagnostic hypothesis of type 2 CPAM.

CT allows for the better evaluation of the lesion and its relationships with adjacent structures. On CT scans, lesions are detectable as well-defined air-filled spaces variable in size depending on CPAM type [15]: type 1 CPAM appears on CT as a one or multiple large cystic structures which can be entirely filled with air or with air-fluid levels (Figure 3, Figure 5 and Figure 6); type 2 CPAMs are visualized on CT as air-filled multicystic masses or focal or ill-defined areas of consolidation [3,15] (Figure 4 and Figure 7); type 3 typically exhibits a solid appearance on CT [15]. In infected CPAMs, imaging can demonstrate internal air-fluid levels and an enhanced thick wall. 

**Figure 8:**
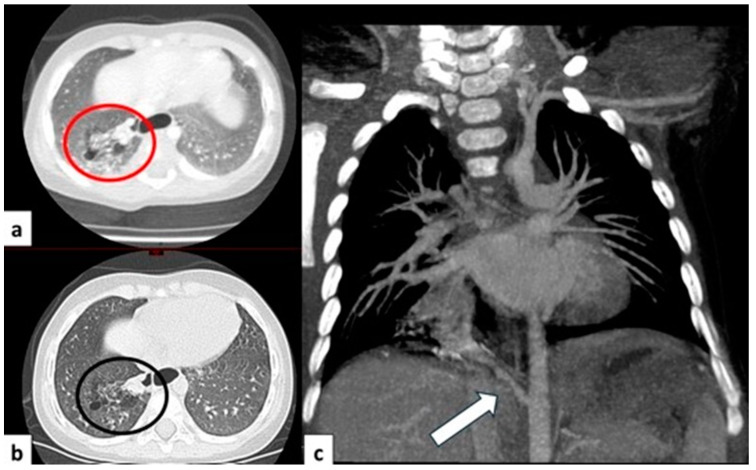
CT axial image visualized on lung window (**a**) of a 9 month old boy with right cystic lesions (previously seen on fetal ultrasound) shows an area of parenchymal consolidation mixed with air-filled cystic lesions, located in the right lower lobe (red circle). Contrast-enhanced acquisitions (**c**) demonstrate an abnormal arterial supply from systemic circulation into sequestered lung (arrow). These findings are compatible with hybrid lesion (CPAM and pulmonary sequestration). A CT scan performed after the arterial embolization of the aberrant vessel (**b**) shows a mild reduction in sequestered lung parenchyma (black circle).

**Figure 9:**
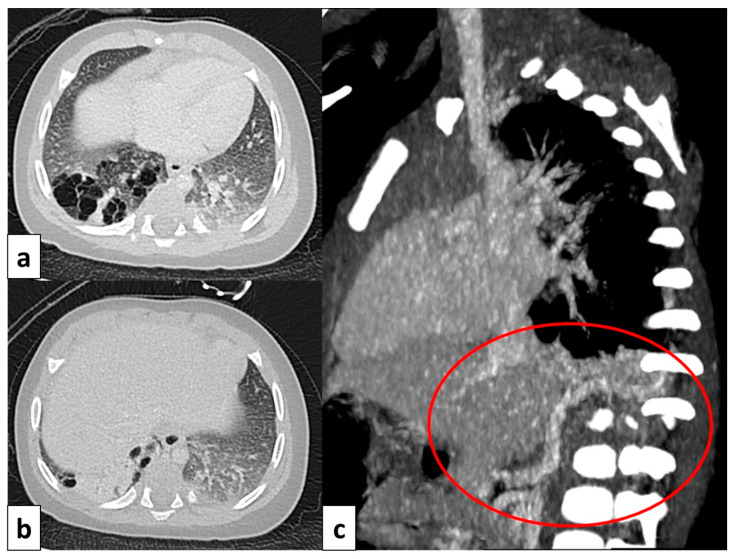
CT image of a 4 month old girl presenting with multiple right cystic lesions <2 cm (**a**) compatible with CPAM type 2. Parenchymal consolidation (**b**) associated with the aberrant arterial (**c**) supply from the celiac trunk (red circle) and venous drainage into the pulmonary venous system, further evaluated with the coronal MiP reconstruction, represent a pulmonary intra-lobar sequestration. The combination of these findings supports the diagnosis of a hybrid lesion.

CT angiography may be useful in differentiating CPAMs from sequestration (which may exhibit cystic features) by excluding abnormal systemic arterial supply in CPAM. However, CPAM and sequestration may coexist, presenting as a hybrid lesion [15] (Figure 8 and Figure 9).

ACPAM’s MRI features are closely tied to the composition of the lesions, which includes the ratio of cystic and solid components, as well as the contents of the cysts. The internal cyst signal is uniformly hypointense on all MR sequences when it is entirely air-filled. Conversely, if the cyst contains fluid, it will typically appear as hyperintense on T2-weighted images [3,32].

#### 3.4.3. Congenital Lobar Hyperinflation

Congenital lobar hyperinflation (CLH), or congenital lobar emphysema, results from progressive lobar overexpansion due to abnormal bronchial cartilage. CLH shows overdistended alveoli with an intact wall and the compression of adjacent lung parenchyma; therefore, the term emphysema is technically inadequate [3,15,32]. CLH shows a clear preference for specific lobes, with the left upper lobe being the most frequently affected (42.2%) [3]. Occasionally, only a segment of a lobe or multiple lobes may be affected [15]. Typically, patients experience respiratory distress symptoms in the first 6 months of life, and CLH diagnosis is achieved during the neonatal period [42]. However, often, CLH is detected “in utero”, appearing as a hypoechoic mass on prenatal US and as a homogeneous mass with increased T2 signal on fetal MRI [3]. 

Following birth, CLH typically manifests a characteristic temporal pattern on neonatal chest radiographs [32]. Within the first hours to days of life, chest radiographs may reveal an area of increased opacity in the lung, attributed to fetal fluid retention immediately after birth [8,15]. As fetal lung fluid is replaced by air, the affected lung gradually becomes hyperlucent on conventional radiographs, and a certain degree of mass effect could be appreciated on the adjacent lobes [3,15]. 

CT scans show a hyperinflated lobe with attenuated and displaced vessels; the latter finding could be useful to achieve a differential diagnosis from pneumothorax or pulmonary cyst [3]. Associated findings may include mediastinal shift, the widening of rib spaces, the depression of the hemidiaphragm, and adjacent compressive atelectasis [3,15,35]. Additionally, CT scans allow for the exclusion of other causes of lobar over-inflation, such as extrinsic bronchial obstruction [37].

#### 3.4.4. Congenital Bronchial Atresia

Congenital bronchial atresia occurs when a lobar, segmental, or subsegmental bronchus is either absent or obstructed, while the distal airway develops normally [15,43]. The bronchi beyond the obstructed segment become dilated and filled with mucus, leading to the formation of a bronchocele. Additionally, the lung segment ahead of the obstruction may become hyperinflated as a consequence of the obstructed passage of air through the interstitium and Kohn’s pores [44]. Even though the precise cause of bronchial atresia remains unclear, two pathogenic theories have been proposed. One theory suggests that bronchial atresia results from a localized absence of connection between the tip of the bronchial bud and primitive bronchial cells, with normal growth distal to the defect [3,32,44]. The other theory is that bronchial atresia occurs due to the interruption of the bronchial arterial supply to a segment of the bronchial wall occurring in intrauterine life, leading to bronchial disconnections caused by ischemia [32,44]. 

Bronchial atresia exhibits a strong association with various congenital anomalies [32]. A study revealed a striking prevalence of bronchial atresia in pulmonary sequestration (100% of cases) and intra-lobar sequestrations (82%). Notably, bronchial atresia was also observed in 70% of CPAMs and 50% of CLH cases [45]. These findings support the pathogenetic theory of CLMs as a “continuum” of lung maldevelopment [46].

Affected patients may manifest symptoms in their childhood, exhibiting respiratory difficulty or repeated infections, which are predictors of associated parenchymal disease. However, the majority of these lesions may be asymptomatic until adulthood and incidentally diagnosed on chest radiographs obtained for other indications [3,15,37]. 

**Figure 10:**
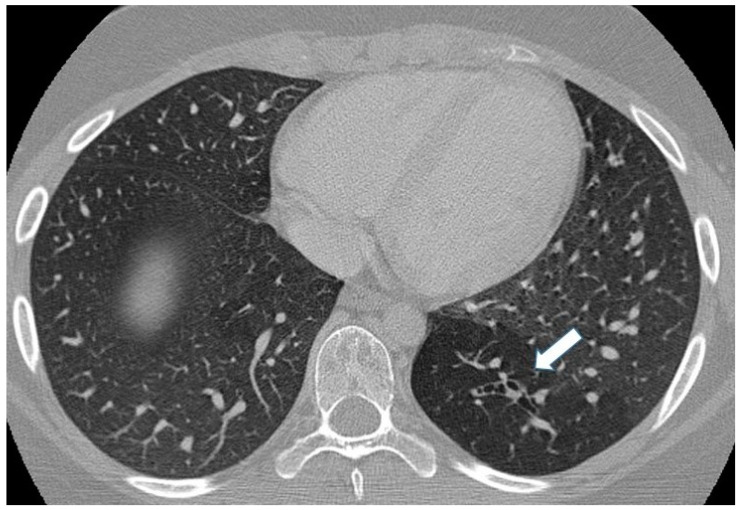
Incidental finding of bronchial atresia during a CT scan in a 15-year-old girl with lymphoma. In the left lower lobe, a tubular-shaped opacity (mucoid-filled bronchus) is associated with segmental hypoattenuation (arrow).

On chest radiographs, mucoceles are frequently visualized as linear, ovoid, or branched opacities, sometimes exhibiting air-fluid levels. Distal air-trapping presents as a focal area of increased lucency [3,47]. CT images demonstrates a tubular-shaped opacity (mucoid-filled bronchus) associated with segmental hypoattenuation and decreased vascularity (due to hyperinflation), mostly located in the apical or apico-posterior segments of the upper lobes, which can be clearly detected during the expiratory phase [15,47] (Figure 10).

In the absence of a mucocele, it may be difficult to differentiate this malformation from CLH.

Unlike in CLH, where the airway is narrowed leading to a valve effect, in bronchial atresia, the airway is completely occluded; hence, the valve effect is absent. As a result, mediastinal shift and the collapse of the ipsilateral lobes, which are commonly found in CLH, are less frequent in bronchial atresia [37,48].

#### 3.4.5. Bronchogenic Cysts

Bronchogenic cysts (BCs) arise from developmental anomalies stemming from abnormal ventral budding or the branching of the tracheobronchial tree, occurring between the 26th and 40th days of fetal life [3,47] Subcarinal lesions predominate, followed by those in the right paratracheal region, para-esophageal area, hilar region, suprasternal notch, and others in miscellaneous locations [3,49]. Additionally, they can develop within pulmonary parenchyma, with the majority located in the lower lobes [47]. BCs are frequently asymptomatic, although in newborns, large-volume cysts can lead to respiratory distress, cyanosis, and feeding difficulties [49]. In adults, symptoms may include chest pain and dysphagia [15].

**Figure 11:**
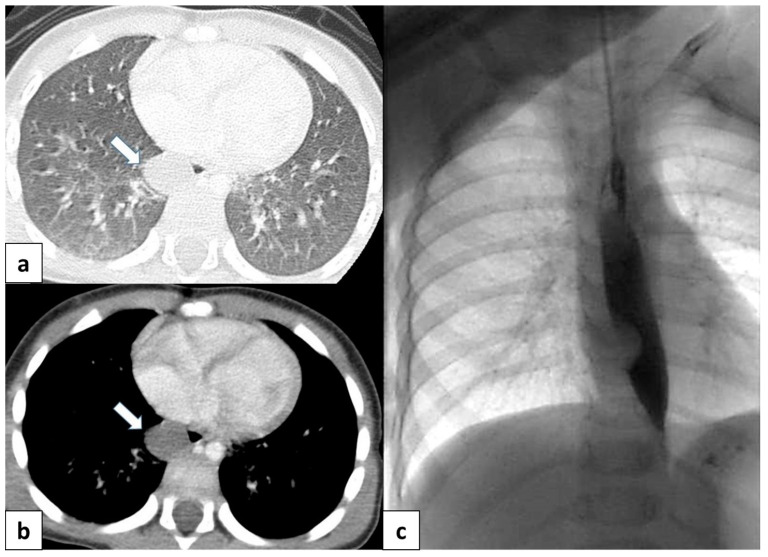
(**a**) CT scan in a 6 month old child shows a well-defined, rounded lesion with uniform fluid attenuation (arrow), compatible with a bronchogenic cyst; (**b**) after administration of intravenous contrast, bronchogenic cyst shows no enhancement or a minimally enhancing wall (arrow). (**c**) Dynamic esophageal fluoroscopy with oral contrast administration revealed a round, filling defect within the esophageal lumen, indicating that the cyst arises extrinsically from the bronchial tree.

BCs can be incidentally detected on chest X-rays and represent approximately 10% of pediatric cases of mediastinal lesions [49]. On chest radiography, these lesions have a nonspecific appearance as water or a dense soft tissue mass mostly located in the mediastinum or central lung [3,15] (Figure 11).

When the suspicion of a BC has been raised on a chest radiograph, a thoracic CT scan is warranted to definitively establish the diagnosis [49]. 

CT helps to evaluate density, the extension of the lesion, and its relationship with the adjacent structure, as well as to differentiate bronchogenic cysts from vascular abnormalities using intravenous contrast [3,15]. CT scans reveal well-defined, smoothly contoured, and rounded lesions with uniform fluid attenuation, although the CTHU attenuation value varies depending on the concentration of proteinaceous elements (Figure 11a,b). Following intravenous contrast administration, bronchogenic cysts typically do not present with signs of enhancement or at most a mildly enhancing wall [15] (Figure 11b).

The presence of an internal air–fluid interface or a markedly thickened wall demonstrating intense contrast enhancement may suggest a secondary infection [15]. 

### 3.5. Vascular Anomalies

#### Pulmonary Arteriovenous Malformation

Pulmonary arteriovenous malformations (PAVMs) are vascular anomalies characterized by the presence of a direct, abnormal connection between pulmonary arteries and veins, bypassing the capillary network of the lung [50]. PAVMs were first recognized in association with hereditary hemorrhagic telangiectasia (HHT) in 1938 [51]. HHT, also known as Osler–Weber–Rendu syndrome, is an autosomal dominant disorder characterized by a triad of epistaxis, mucocutaneous or visceral telangiectasia, and a family history of PAVMs. Approximately 70% of PAVMs occur within the context of HHT syndrome, with the remaining 30% classified as sporadic cases [52]. 

PAVMs can be categorized into two main types: simple and complex. Simple PAVMs involve a single communication channel between a pulmonary artery and vein, while complex PAVMs comprise multiple feeding arteries and draining veins [53]. Notably, patients with PAVMs are often asymptomatic, with the presence of symptoms primarily linked to the size of the malformation rather than the number [52]. As the degree of right-to-left shunting increases, patients may progressively develop dyspnea and cyanosis. In severe cases, complications such as paradoxical embolization leading to brain infarction or abscess formation and hemoptysis or hemothorax due to PAVM rupture can occur [47]. On chest radiography, PAVMs usually appear as a well-marginated lesion with concomitant curvilinear opacities, representing arterial inflow and venous outflows toward the hilum, mostly located in the lower lobes [15,52]. They can also have a more complex architecture with dilated vessels forming a plexiform mass [53,54].

**Figure 12:**
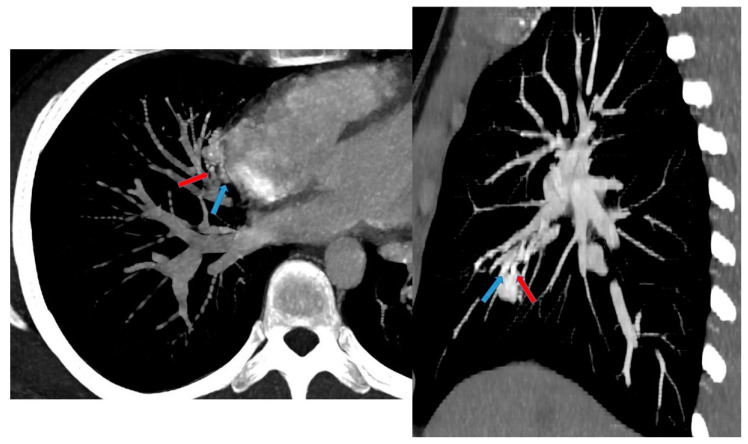
A 17 year old patient with hereditary hemorrhagic telangiectasis (HHT). CT angiography with maximum intensity projection (MIP) reconstruction shows a lobulated enhancing lesion in the right middle lobe, with a feeding artery (red arrow) and a draining vein (blue arrow), compatible with pulmonary arteriovenous malformation.

On non-contrast CT, PAVMs typically can be seen as lobulated or serpiginous intraparenchymal masses with soft tissue attenuation. Following the administration of intravenous contrast, these lesions demonstrate marked enhancement. CT may be helpful in the characterization of AVM with complex angioarchitecture, especially using angiography protocol combined with 3D reconstruction [15,32] (Figure 12).

Other vascular anomalies included in the spectrum of CLMs are summarized in Table 2.

### 3.6. Combined Lung and Vascular Anomalies

#### 3.6.1. Pulmonary Sequestration

Pulmonary sequestration (PS) is second to CPAMs in terms of frequency among congenital lung malformations [3]. It is characterized by a dysplastic area of lung parenchyma that lacks a direct connection to the tracheobronchial tree and receives its blood supply from the extra-pulmonary circulation [3,15]. The arterial supply typically originates from the thoracic or abdominal aorta. However, cases have been documented where the arterial supply arises from a variety of atypical sources, including the intercostal, subclavian, celiac, splenic, and coronary arteries [3,55].

PS can be classified into two distinct types based on the relationship of the anomalous lung segment to the pleura and its venous drainage: intralobar and extralobar sequestration [3,15,32]. The extra-lobar form has its own pleural investment and systemic venous drainage. The theory that this form represents a congenital anomaly is generally accepted, and it accounts for 25% of sequestration cases [3,5,8,15,55]. In contrast, the intra-lobar form (75% of cases) manifests within the lung but is not covered by its own pleura and usually drains into the pulmonary venous system [3,15]. A growing base of data about the underlying cause of intra-lobar sequestrations supports the idea that intra-lobar sequestration stems from recurrent infections that produce aberrant arterial vessels arising from the aorta [15,35]. 

The presenting features of pulmonary sequestrations vary depending on the specific subtype. Intra-lobar sequestrations typically manifest in older children or adults with recurrent respiratory infections localized to the affected lung segment. In contrast, extra-lobar sequestrations typically present in the prenatal/neonatal period as focal masses, causing—depending on lesion size and associated congenital lung anomalies—symptoms such as respiratory distress and cyanosis [15]. Extra-lobar sequestrations are frequently associated with other congenital malformations, occurring in up to 65% of cases. These associated anomalies can include CPAM, congenital diaphragmatic hernia or other diaphragmatic malformations, bronchogenic cyst, foregut duplication cysts, bronchial atresia, pulmonary hypoplasia, and scimitar syndrome [56]. The primary features of both intra-lobar and extra-lobar sequestrations are reported in Table 3.

**Figure 13:**
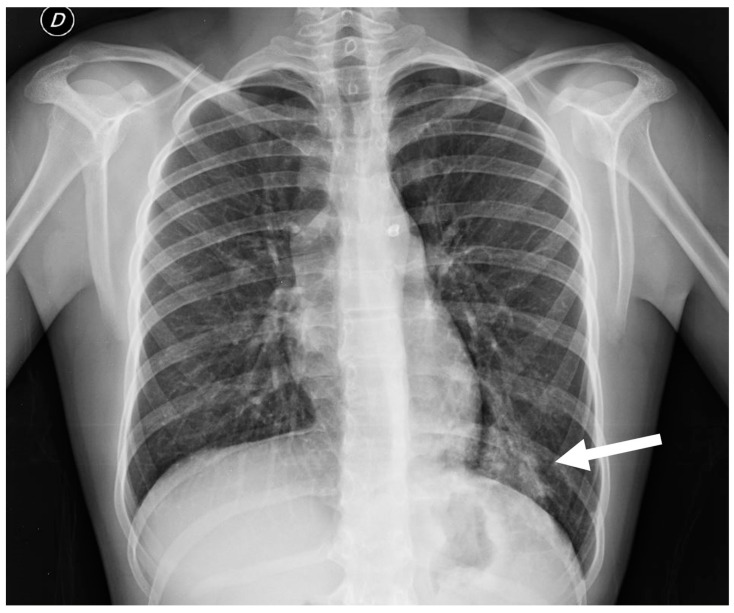
Chest radiograph of a 17 year old boy shows focal lung masses within the left lower lobe with microcystic appearance due to recurrent infection, suggesting an intra-lobar sequestration (arrow).

Chest radiography is a valuable initial imaging modality for pulmonary sequestrations. In up to 98% of cases, it demonstrates these malformations as circumscribed lung masses predominantly involving the lower lobes, with a left-sided predilection [15,35]. Additionally, the anomalous systemic arterial supply may sometimes be visualized on plain radiographs [4]. In cases of intra-lobar sequestration, repetitive infections can predispose a patient to liquefaction necrosis, manifesting radiographically as a mainly cystic lesion that may contain air-fluid levels [8,15] (Figure 13).

**Figure 14:**
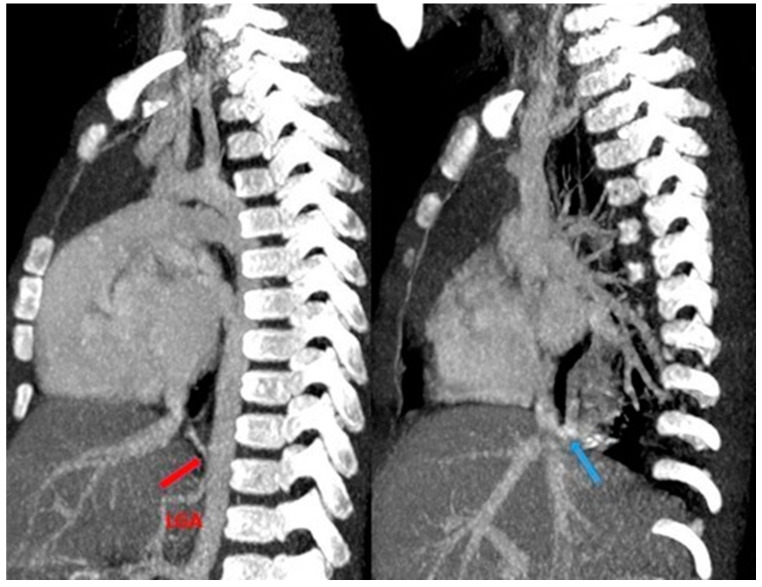
CT angiography with maximum intensity projection (MIP) reconstruction shows an anomalous artery (red arrow) arising from left gastric artery (LGA) into sequestered lung and an anomalous vein (blue arrow) draining into inferior vena cava, in its intrahepatic tract. These findings are compatible with extra-lobar sequestration.

**Figure 15:**
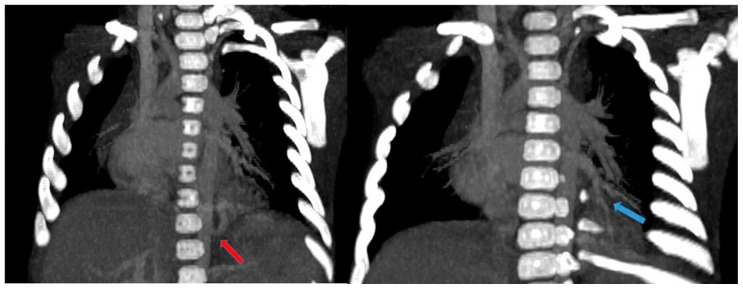
CT angiography with maximum intensity projection (MIP) reconstruction shows an anomalous artery (red arrow) arising from abdominal aorta and coursing toward the sequestered lung and an anomalous vein (blue arrow) draining into the pulmonary venous system. These findings are compatible with intra-lobar sequestration.

**Figure 16:**
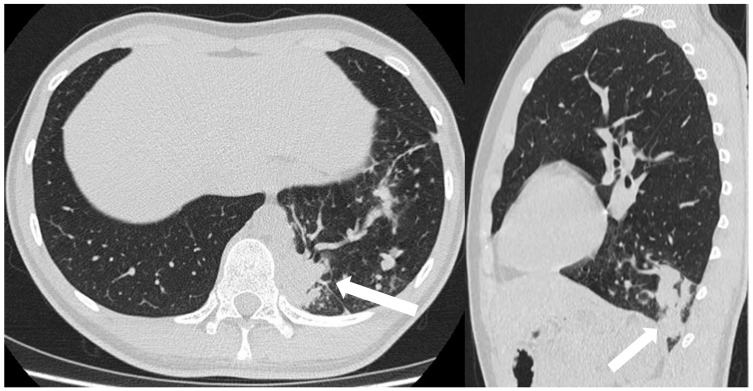
CT scans in axial and sagittal view of a 17 year old boy with suspected intra-lobar sequestration show a homogeneous soft tissue density mass within lung parenchyma (arrows).

CT angiography with 3D reconstruction serves as a highly informative tool for identifying anomalous feeding arteries and drainage veins for differentiating between intra- and extra-lobar sequestration (Figure 14 and Figure 15). The CT appearance of the lesion ranges from a homogeneous soft tissue mass within the parenchyma (Figure 16) to a cystic lesion containing air or fluid, which can coexist in hybrid forms [15,37].

#### 3.6.2. Hypogenetic Lung Syndrome

Hypogenetic lung syndrome, also known as scimitar syndrome, is a pathological condition in which an hypoplastic right lung has an anomalous pulmonary venous drainage associating with the dextroposition of the heart and anomalous systemic arterial supply to the right lung [3,15]. The anomalous venous return is usually into the inferior vena cava but others have reported an anomalous drainage into the portal vein, hepatic vein, superior vena cava, right atrium, and azygos vein [3]. Subsequently, there is a left-to-right shunt, due to the anomalous pulmonary venous drainage [3,32]. The clinical presentation depends on the degree of anomalous shunt and the age of patients: small shunts often remain asymptomatic for many years, particularly in older children. However, patients may eventually develop symptoms such as fatigue, dyspnea, or recurrent pulmonary infections. Conversely, large shunts in infants can lead to significant clinical manifestations. Right heart failure due to volume overload and pulmonary hypertension are potential complications arising from the increased blood flow to the lungs [3,32].

Postnatal chest radiography may reveal characteristic findings suggestive of scimitar syndrome. These include an anomalous pulmonary vein manifesting as a curved opacity in the right lower lobe, coursing toward the right hemidiaphragm and resembling a scimitar sword. Additionally, the right lung exhibits hypoplasia and hyperlucency, with reduced lung volume and rightward mediastinal shift. Conversely, the left lung often exhibits compensatory hyperinflation [3,4,8].

**Figure 17:**
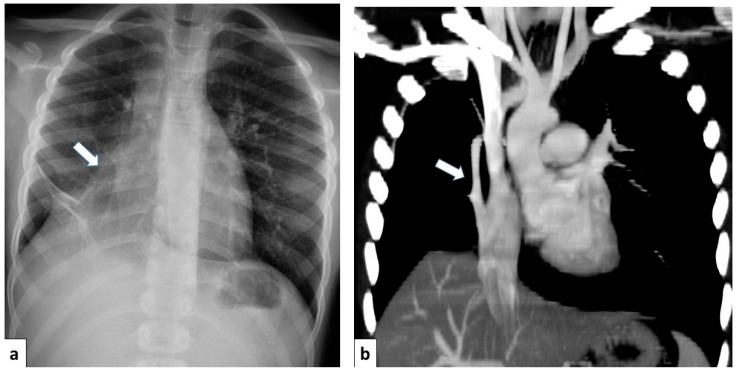
(**a**) Chest radiograph of a 11 year old boy shows an anomalous vein appearing as a curvilinear opacity (arrow) in the right lower lobe, similar to a scimitar sword; note that the right lung is hypoplastic; (**b**) CT angiography with coronal MIP reconstruction demonstrates the entirety of the anomalous scimitar vein and its associated drainage in the inferior vena cava (arrow).

CT angiography with 3D reconstruction is able to evaluate the entirety of the anomalous scimitar vein and its associated drainage site [4] (Figure 17).

## 4. Conclusions

Although the pathological classification of CLMs requires histological assessment, it is useful to provide a general classification and a description of each phenotypic feature because more than one of these anomalies may be present in the same case. CLMs lead to significant morbidity and mortality, so a systematic diagnostic approach may permit the early identification of these anomalies and their optimal prenatal counseling and post-natal management.

## Figures and Tables

**Table 1:** Classification and features of congenital pulmonary airway malformations.

	Type 0	Type 1	Type 2	Type 3	Type 4
** Incidence **	2%	60–65%	10–40%	5–10%	10–15%
** Origin **	tracheo-bronchial	bronchial	bronchiolar	alveolar duct	distal acinar
** Imaging **	acinar dysgenesis or dysplasia; cardiovascular anomalies	large cystic lesions (2–10 cm) with small cysts around	multiple small cysts (0.5–2 cm). cardiovascular and renal malformation may be associated	“adenomatoid”: small cystic lesions (<5 mm) with solid aspect	large multilocular cysts (>10 cm) at the lobe’s periphery
** Clinical Presentation **	incompatible with life	asymptomatic, respiratory distress or infection	asymptomatic, respiratory distress or infection	prenatal hydrops, postnatal respiratory distress	incidental findings

**Table 2:** Other vascular anomalies within the spectrum of CLMs.

	Characteristics
** Proximal Interruption of the Pulmonary Artery **	the affected pulmonary artery terminates within 1 cm of its origin from the main pulmonary artery with systemic collateral vessels supplying the involved lung that is hypoplastic.
** Pulmonary Artery Sling **	the left main pulmonary artery arises from the proximal right main pulmonary artery and courses to the left hemithorax behind the carina
** Pulmonary Vein Stenosis **	thickening and narrowing of the pulmonary veins usually occurred at the pulmonary venous and left atrial junction.

**Table 3:** Main features of intra-lobar and extra-lobar sequestration.

	Intralobar Type	Extralobar Type
** Incidence **	75%	25%
** Cause **	acquired or congenital	congenital
** Timing of Diagnosis **	childhood or adulthood	Prenatal–neonatal period
** Pleural Investment **	within lobe without its own pleura	with its own lung pleura
** Arterial Supply **	thoracic or abdominal aorta	abdominal aorta
** Venous Drainage **	pulmonary venous system	systemic venous system
** Association with Other Congenital Anomalies **	rare	common

## Data Availability

Not applicable.

## References

[1] Annunziata F., Bush A., Borgia F., Raimondi F., Montella S., Poeta M., Borrelli M., Santamaria F. (2019). Congenital Lung Malformations: Unresolved Issues and Unanswered Questions. Front. Pediatr..

[2] Stocker L.J., Wellesley D.G., Stanton M.P., Parasuraman R., Howe D.T. (2015). The increasing incidence of foetal echogenic congenital lung malformations: An observational study. Prenat. Diagn..

[3] Biyyam D.R., Chapman T., Ferguson M.R., Deutsch G., Dighe M.K. (2010). Congenital lung abnormalities: Embryologic features, prenatal diagnosis, and postnatal radiologic-pathologic correlation. Radiographics.

[4] Zylak C.J., Eyler W.R., Spizarny D.L., Stone C.H. (2002). Developmental lung anomalies in the adult: Radiologic-pathologic correlation. RadioGraphics.

[5] Panicek D.M., Heitzman E.R., Randall P.A., Groskin S.A., Chew F.S., Lane E.J., Markarian B. (1987). The continuum of pulmonary developmental anomalies. Radiographics.

[6] Boucherat O., Jeannotte L., Hadchouel A., Delacourt C., Benachi A. (2016). Pathomechanisms of congenital cystic lung diseases: Focus on congenital cystic adenomatoid malformation and pleuropulmonary blastoma. Paediatr. Respir. Rev..

[7] Seear M., Townsend J., Hoepker A., Jamieson D., McFadden D., Daigneault P., Glomb W. (2017). A review of congenital lung malformations with a simplified classification system for clinical and research use. Pediatr. Surg. Int..

[8] Lee E.Y., Dorkin H., Vargas S.O. (2011). Congenital pulmonary malformations in pediatric patients: Review and update on etiology, classification, and imaging findings. Radiol. Clin. N. Am..

[9] Swarr D.T., Peranteau W.H., Pogoriler J., Frank D.B., Adzick N.S., Hedrick H.L., Morley M., Zhou S., Morrisey E.E. (2018). Novel molecular and phenotypic insights into congenital lung malformations. Am. J. Respir. Crit. Care Med..

[10] Feinberg A., Hall N.J., Williams G.M., Schultz K.A., Miniati D., Hill D.A., Dehner L.P., Messinger Y.H., Langer J.C. (2016). Can congenital pulmonary airway malformation be distinguished from Type I pleuropulmonary blastoma based on clinical and radiological features?. J. Pediatr. Surg..

[11] Mon R.A., Johnson K.N., Ladino-Torres M., Heider A., Mychaliska G.B., Treadwell M.C., Kunisaki S.M. (2019). Diagnostic accuracy of imaging studies in congenital lung malformations. Arch. Dis. Child. Fetal Neonatal Ed..

[12] Laberge J.M., Flageole H., Pugash D., Khalife S., Blair G., Filiatrault D., Russo P., Lees G., Wilson R. (2001). Outcome of the prenatally diagnosed congenital cystic adenomatoid lung malformation: A Canadian experience. Fetal Diagn. Ther..

[13] Bush A., Hogg J., Chitty L.S. (2008). Cystic lung lesions—Prenatal diagnosis and management. Prenat. Diagn..

[14] Baird R., Puligandla P.S., Laberge J.M. (2014). Congenital lung malformations: Informing best practice. Semin. Pediatr. Surg..

[15] Lee E.Y., Boiselle P.M., Cleveland R.H. (2008). Multidetector CT evaluation of congenital lung anomalies. Radiology.

[16] Bush A. (2015). Rare Lung Diseases: Congenital Malformations. Indian J. Pediatr..

[17] Hermelijn S.M., Zwartjes R.R., Tiddens H.A.W.M., Cochius-den Otter S.C.M., Reiss I.K.M., Wijnen R.M.H., Schnater J.M. (2020). Associated Anomalies in Congenital Lung Abnormalities: A 20-Year Experience. Neonatology.

[18] Montella S., Corcione A., Santamaria F. (2017). Recurrent Pneumonia in Children: A Reasoned Diagnostic Approach and a Single Centre Experience. Int. J. Mol. Sci..

[19] Cherian S.V., Kumar A., Ocazionez D., Estrada-Y-Martin R.M., Restrepo C.S. (2019). Developmental lung anomalies in adults: A pictorial review. Respir. Med..

[20] Trotman-Dickenson B. (2015). Congenital lung disease in the adult: Guide to the evaluation and management. J. Thorac. Imaging.

[21] Kotecha S., Barbato A., Bush A., Claus F., Davenport M., Delacourt C., Deprest J., Eber E., Frenckner B., Greenough A. (2012). Antenatal and postnatal management of congenital cystic adenomatoid malformation. Paediatr. Respir. Rev..

[22] Lakhoo K. (2009). Management of congenital cystic adenomatous malformations of the lung. Arch. Dis. Child. Fetal Neonatal Ed..

[23] Priest J.R., Williams G.M., Hill D.A., Dehner L.P., Jaffé A. (2009). Pulmonary cysts in early childhood and the risk of malignancy. Pediatr. Pulmonol..

[24] Nasr A., Himidan S., Pastor A.C., Taylor G., Kim P.C. (2010). Is congenital cystic adenomatoid malformation a premalignant lesion for pleuropulmonary blastoma?. J. Pediatr. Surg..

[25] Messinger Y.H., Stewart D.R., Priest J.R., Williams G.M., Harris A.K., Schultz K.A., Yang J., Doros L., Rosenberg P.S., Hill D.A. (2015). Pleuropulmonary blastoma: A report on 350 central pathology-confirmed pleuropulmonary blastoma cases by the International Pleuropulmonary Blastoma Registry. Cancer.

[26] Rossi G., Gasser B., Sartori G., Migaldi M., Costantini M., Mengoli M.C., Piccioli S., Cavazza A., Rivasi F. (2012). MUC5AC, cytokeratin 20 and HER2 expression and K-RAS mutations within mucinogenic growth in congenital pulmonary airway malformations. Histopathology.

[27] Lantuejoul S., Nicholson A.G., Sartori G., Piolat C., Danel C., Brabencova E., Goldstraw P., Brambilla E., Rossi G. (2007). Mucinous cells in type 1 pulmonary congenital cystic adenomatoid malformation as mucinous bronchioloalveolar carcinoma precursors. Am. J. Surg. Pathol..

[28] Casagrande A., Pederiva F. (2016). Association between Congenital Lung Malformations and Lung Tumors in Children and Adults: A Systematic Review. J. Thorac. Oncol..

[29] Girsen A.I., Hintz S.R., Sammour R., Naqvi A., El-Sayed Y.Y., Sherwin K., Davis A.S., Chock V.Y., Barth R.A., Rubesova E. (2019). Prediction of neonatal respiratory distress in pregnancies complicated by fetal lung masses. Prenat. Diagn..

[30] Ruchonnet-Metrailler I., Leroy-Terquem E., Stirnemann J., Cros P., Ducoin H., Hadchouel A., Khen-Dunlop N., Labbé A., Labouret G., Lebras M.N. (2014). Neonatal outcomes of prenatally diagnosed congenital pulmonary malformations. Pediatrics.

[31] Shulman R., Sparks T.N., Gosnell K., Blat C., Norton M.E., Lee H., Gonzalez-Velez J., Goldstein R.B. (2019). Fetal Congenital Pulmonary Airway Malformation: The Role of an Objective Measurement of Cardiomediastinal Shift. Am. J. Perinatol..

[32] Thacker P.G., Rao A.G., Hill J.G., Lee E.Y. (2014). Congenital lung anomalies in children and adults: Current concepts and imaging findings. Radiol. Clin. N. Am..

[33] Chen H.W., Hsu W.M., Lu F.L., Chen P.C., Jeng S.F., Peng S.S., Chen C.Y., Chou H.C., Tsao P.N., Hsieh W.S. (2010). Management of congenital cystic adenomatoid malformation and bronchopulmonary sequestration in newborns. Pediatr. Neonatol..

[34] Bush A. (2009). Prenatal presentation and postnatal management of congenital thoracic malformations. Early Hum. Dev..

[35] Berrocal T., Madrid C., Novo S., Gutiérrez J., Arjonilla A., Gómez-León N. (2004). Congenital anomalies of the tracheobronchial tree, lung, and mediastinum: Embryology, radiology, and pathology. Radiographics.

[36] Yancey M.K., Richards D.S. (1993). Antenatal sonographic findings associated with unilateral pulmonary agenesis. Obstet. Gynecol..

[37] Garcia-Peña P., Coma A., Enríquez G. (2013). Congenital lung malformations: Radiological findings and clues for differential diagnosis. Acta Radiol..

[38] Quinn T.M., Hubbard A.M., Adzick N.S. (1998). Prenatal magnetic resonance imaging enhances fetal diagnosis. J. Pediatr. Surg..

[39] Mata J.M., Cáceres J. (1996). The dysmorphic lung: Imaging findings. Eur. Radiol..

[40] Stocker J.T., Madewell J.E., Drake R.M. (1977). Congenital cystic adenomatoid malformation of the lung: Classification and morphologic spectrum. Hum. Pathol..

[41] Stocker J.T. (2002). Congenital pulmonary airway malformation: A new name for and an expanded classification of congenital cystic adenomatoid malformation of the lung. Symposium 24: Non-neoplastic lung disease. Histopathology.

[42] Paterson A. (2005). Imaging evaluation of congenital lung abnormalities in infants and children. Radiol. Clin. N. Am..

[43] Webb W., Higgins C.B., Webb W.R. (2005). Congenital bronchopulmonary lesions. Thoracic Imaging: Pulmonary and Cardiovascular Radiology.

[44] Fraser R., Colman N., Muller N.L., Pare P.D., Fraser R., Colman N., Muller N.L., Pare P.D. (2005). Developmental and metabolic lung disease. Synopsis of Diseases of the Chest.

[45] Riedlinger W.F., Vargas S.O., Jennings R.W., Estroff J.A., Barnewolt C.E., Lillehei C.W., Wilson J.M., Colin A.A., Reid L.M., Kozakewich H.P.W. (2006). Bronchial atresia is common to extralobar sequestration, intralobar sequestration, congenital cystic adenomatoid malformation, and lobar emphysema. Pediatr. Dev. Pathol..

[46] Langston C. (2003). New concepts in the pathology of congenital lung malformations. Semin. Pediatr. Surg..

[47] Watarai F., Takahashi M., Hosoya T., Murata K. (2012). Congenital lung abnormalities: A pictorial review of imaging findings. Jpn. J. Radiol..

[48] Castellote A., Enriquez G., Lucaya J., Cathy H., Brunelle P., Stringer D.A., Kao S.C.S. (2005). Congenital malformations of the chest beyond the neonatal period. Imaging Children.

[49] Nadeem M., Elnazir B., Greally P. (2012). Congenital pulmonary malformation in children. Scientifica.

[50] Gossage J.R., Kanj G. (1998). Pulmonary arteriovenous malformations. A state of the art review. Am. J. Respir. Crit. Care Med..

[51] Goodenberger D.M., Chakinala M., Grippi M.A., Elias J.A., Fishman J.A., Kotloff R.M., Pack A.I., Senior R.M., Siegel M.D. (2015). Pulmonary arteriovenous malformations. Fishman’s Pulmonary Diseases and Disorders.

[52] Tellapuri S., Park H.S., Kalva S.P. (2019). Pulmonary arteriovenous malformations. Int. J. Cardiovasc. Imaging.

[53] White R.I., Mitchell S.E., Barth K.H., Kaufman S.L., Kadir S., Chang R., Terry P.B. (1983). Angioarchitecture of pulmonary arteriovenous malformations: An important consideration before embolotherapy. AJR Am. J. Roentgenol..

[54] Primack S.L., Müller N.L., Mayo J.R., Remy-Jardin M., Remy J. (1994). Pulmonary parenchymal abnormalities of vascular origin: High-resolution CT findings. Radiographics.

[55] Newman B. (2006). Congenital bronchopulmonary foregut malformations: Concepts and controversies. Pediatr. Radiol..

[56] Conran R.M., Stocker J.T. (1999). Extralobar sequestration with frequently associated congenital cystic adenomatoid malformation, type 2: Report of 50 cases. Pediatr. Dev. Pathol..

